# Nitrogen Supply Alters Rice Defense Against the Striped Stem Borer *Chilo suppressalis*

**DOI:** 10.3389/fpls.2021.691292

**Published:** 2021-07-26

**Authors:** Yueqin Zheng, Xiyong Zhang, Xin Liu, Ningning Qin, Kaifang Xu, Rensen Zeng, Jian Liu, Yuanyuan Song

**Affiliations:** ^1^Key Laboratory of Ministry of Education for Genetics, Breeding and Multiple Utilization of Crops, College of Life Sciences, Fujian Agriculture and Forestry University, Fuzhou, China; ^2^Institute of Crop Resistance and Chemical Ecology, College of Agriculture, Fujian Agriculture and Forestry University, Fuzhou, China; ^3^State Key Laboratory of Ecological Pest Control for Fujian and Taiwan Crops, Fujian Agriculture and Forestry University, Fuzhou, China

**Keywords:** constitutive defense, induced defense, jasmonic acid, lignin, metabolome, nitrate, rice, striped stem borer

## Abstract

Plant nutrition status is closely associated with plant defense against insect herbivores. However, the way nitrogen supply regulates rice anti-herbivore is not clear. This study investigated the effects of low (LN, 0.3 mM) and high (HN, 3 mM) nitrate levels on rice resistance against the striped stem borer *Chilo suppressalis* (SSB), one of the major destructive rice pests. Seven-day-old rice seedlings were cultured with different nitrate levels for 30 days and then inoculated with third instars of SSB. LN significantly enhanced rice anti-herbivore defense and lowered the total nitrogen content in the plants, but increased the content of free amino acids after SSB infestation. Additionally, LN significantly increased the accumulation of phenolic acids and flavonoids, especially lignin, resulting in enhanced constitutive defense in SSB-infested plants. SSB feeding led to a rapid accumulation of secondary metabolites. HN application led to the accumulation of metabolites derived from cinnamic acid, *p*-coumaric acid, *p*-coumaric CoA, feruloyl CoA, and apigenin, while LN led to the accumulation of metabolites derived from 3-dehydroquinic acid, phenylalanine, acetyl CoA, and aspartic acid. Collectively, our finding suggests that nitrogen deficiency enhances rice anti-herbivore defense *via* constitutive defense by the accumulation of phenolic acids and flavonoids.

## Introduction

In response to herbivore attacks, plants have evolved a wide spectrum of strategies to defend themselves against herbivores, such as constitutive defense and induced defense (Tiffin, 2000). Constitutive defense is always expressed, whereas induced defense is activated only after plants are attacked by herbivores (Kempel et al., 2011). Many evolutionary models of induced defense treat it as being derived from constitutive defense, the presumed ancestral state (Thaler and Karban, 1997). Trade-offs between constitutive defense and induced defense with and among species are likely to be beneficial to plants (Morris et al., 2006; Zhang et al., 2008). The constitutive defense and induced defense are both influenced by environmental factors and closely associated with plant physiological characteristics, nutritional status, and the accumulation of secondary metabolites.

Plants produce a tremendous number of secondary metabolites to defend against herbivores (Mumm and Hilker, 2006). Herbivory by *Helicoverpa zea* induced great changes in precursor amino acids in the shikimate pathway in tomato (Steinbrenner et al., 2011). Shikimate-derived amino acids and simple phenylpropanoids are precursors for many secondary metabolites (Herrmann and Weaver, 1999). In these compounds, flavonoids and phenolics are known to be effective defensive compounds against herbivores (Sampedro et al., 2011). Toxins such as flavonoids are considered primarily effective against generalist pests (Diaz Napal et al., 2010). Putative phenolic acid derivatives were also identified as important metabolites produced by plants during antibacterial defense mechanisms (Luzzatto et al., 2007).

Lignin is one of the most important phenolic acids, providing plants with physical and chemical defense mechanisms against herbivores (Liu Q. et al., 2018). Lignin serves as an important barrier that protects plants against herbivory. In rice, treatment with an insect-specific toxin peptide LqhIT2 enhanced the lignin content, leading to enhanced resistance to leafroller (Tianpei et al., 2015). In the pre-ingestion phase, host plants can limit food supplies to insects *via* physical barriers such as the cell wall fortification. Lignin and other phenolics can strengthen cell walls against digestion (Brodeur-Campbell et al., 2006; Schroeder et al., 2006). In addition, increased lignin deposition might have additional negative effects on insect fitness because phenoloxidase enzymes are involved in lignin polymerization as well as in the generation of toxic by-products such as reactive oxygen species, quinones, and peroxides (Felton et al., 1989; Stevenson et al., 1993).

Plants grown with limited resources may produce more phenolic compounds but show slow growth (Wilkens et al., 1996). Nitrogen is an essential macronutrient and a major limiting factor of plant growth and development. Besides, nitrogen can also impact the ability of plants to cope with biotic stress (Ballini et al., 2013). For example, high nitrogen fertilization enhances *Botrytis cinerea* in strawberry (Daugaard et al., 2003), while it reduces susceptibility to this fungus in tomato (Vega et al., 2015). Additionally, the form of nitrogen available can also determine the effect of nitrogen supply on plant response to biotic stress. For instance, ammonium supply reduces the resistance to *Pseudomonas syringae*, while nitrate supply enhances plant resistance to this bacterium (Mur et al., 2017).

Nitrogen content and form play a vital role in defensive primary and secondary metabolism. It can influence defense *via* amino acid metabolism and hormone production. Nitrogen may have negative effects on physical defenses and the production of phytoalexins, but positive effects on defense-related enzymes and proteins to affect local and systemic defense mechanisms (Sun Y. et al., 2020). In most of the rice-growing areas, the increasing populations of major insect pests of rice are closely related to the long-term excessive application of nitrogen fertilizers (Lu et al., 2007). Increased numbers of both adult and immature whiteflies occurred with increasing amounts of applied nitrogen (Bi et al., 2001). Increased nitrogen availability in a tomato leads to a decreased allocation to defenses and increased preference of two-spotted spider mite (*Tetranychus urticae*) females (Hoffland et al., 2000). However, the molecular mechanism and the alteration of metabolism during the interaction between plants and insect herbivores under different nitrogen supply are still unclear. Rice (*Oryza sativa* L.) is an important food crop. The striped stem borer (SSB), *Chilo suppressalis* (Walker), is one of the most economically important and destructive rice pests, which is widely distributed in rice-production countries, leading to huge rice yield losses, particularly in China (Sun et al., 2018). This study aimed to examine how nitrogen supply level affects anti-herbivore defense against SSB and metabolome responses to insect herbivory in rice plants.

## Materials and Methods

### Plant Cultivation

Rice (*Oryza sativa* L. cv. Ishikari-shiroge) seeds were surface-sterilized with 10% (v/v) H_2_O_2_ for 15 min and rinsed with distilled water three times. The sterilized seeds were pre-imbibed in distilled water for 1 day in darkness and then transferred to seedling tray for 7 days. After germination, the seedlings were cultured with modified Kimura B nutrient solution containing two concentrations of KNO_3_ (Li et al., 2016). For LN treatment, the nitrogen concentration in nutrient solution was 0.3 mM KNO_3_. For HN treatment, 3 mM KNO_3_ was added in Kimura B nutrient solution. Given the important role of potassium in plant defense and growth, the same concentration of potassium was added in LN group to replenish potassium. The plants were cultured in a growth chamber with a day: night temperature regime of 28°C (14 h): 22°C (10 h) and a light intensity of 30,000 lux for 30 days.

### Striped Stem Borer Treatment

The original eggs of SSB were provided by the State Key Laboratory for Biology of Plant Diseases and Insect Pests, Chinese Academy of Agricultural Sciences. All cultures were kept under the conditions of 27 ± 1°C, a photoperiod of 16:8 (L:D) h, and 70–80% RH, except for adult mating and oviposition at 85–90% RH (Han et al., 2012). Plants cultured for 30 days were infested with third instars of SSB for biochemical analysis or bioassays. The moment the larva started to chew a hole was defined as time point zero for time course experiments. The stems around 3 cm of the entry hole were harvested at different time points after SSB attack (Hu et al., 2018). For the determination of feeding preference of SSB, each group contained 120 plants, and each plant was inoculated with one third-instar larva of SSB. The feeding was counted as the larva chewed a hole on the stem. The number of feeding SSB was recorded every 30 min in the first 4 h (Tong et al., 2012).

### Measurement of Leaf Chlorophyll and Plant Total Nitrogen

The relative chlorophyll content of rice leaves was measured by a chlorophyll meter SPAD-plus 502 (Konica Minolta Camera Co., Ltd., Japan) according to the method previously described by Sun et al. (2019). For the determination of total nitrogen, the plant samples were oven-dried for at least 24 h at 65°C and weighed; then the material was ground. The total nitrogen content of rice seedlings (mg N per g dry weight) was analyzed using a Foss Kjeltec 8400 analyzer (Kjeltec Analyzer Unit, Foss Tecator AB, Hoganas, Sweden) (Liu J. et al., 2018).

### Determination of Free Amino Acids and Soluble Sugars

Total free amino acids (FAAs) were determined using the ninhydrin colorimetric method (Rosen, 1957). Briefly, the total FAAs from rice seedlings were extracted in ethanol/NaAc buffer (pH 5.4) and then measured using a colorimetric assay at 570 nm. The FAA content was calculated on the basis of a calibration curve by 1-Leu. The contents of soluble sugar in rice seedling were determined according to the methods described in the study by Dubois et al. (1951). Briefly, approximately 100 mg of the oven-dried sample was ground and then extracted with water at 95°C for 10 min. The solution was then centrifuged at 8,000 *g* at 25°C for 10 min. The resulting supernatants were combined and analyzed using the anthrone–sulfuric acid method (Kuang et al., 2017).

### Plant Hormone Analysis

Rice seedlings were cultured with different concentrations of nitrates for 30 days. Stems were harvested at 0, 3, 9 or 24 h after SSB infestation. Samples were immediately immersed in liquid nitrogen and stored at −80°C. For each time interval, five plants were sampled. JA, JA-Ile, SA, and abscisic acid (ABA) were extracted for LC-MS analysis using labeled internal standards as described in the study by Pan et al. (2010).

### Determination of Peroxidase and Polyphenol Oxidase Activities

Two defense-related enzymes, namely, determination of peroxidase (POD) (EC 1.11.1.7) and Polyphenol Oxidase (PPO) (EC1.10.3.1), were analyzed. Leaf samples (0.1 g fresh weight) were ground in liquid nitrogen and homogenized in 0.05 M phosphate buffer (pH 7.2 for POD, pH 7.8 for PPO) containing 1% (w/v) polyvinylpyrrolidone (PVP). The supernatant after 12,000 g centrifugation for 15 min at 4°C was used for enzyme assays. Activities of POD and PPO were spectrophotometrically determined according to the previous study (Song et al., 2014). For POD and PPO, the change in absorbance at 470/525 nm by 0.005/0.01 was an enzyme activity unit per minute per gram of tissue in each mL of the reaction system. Three independent biological replicates for each treatment were used for enzyme assays.

### Determination of Lignin Content and Phloroglucinol Staining

Quantitative determination of lignin was measured by the method of Fukushima and Hatfield (2004). Acetyl bromide was analyzed according to the study by Foster et al. (2010). Lignin in the stem was stained as described in the previous study using the Wiesner reagent test (Shan et al., 2008). Stem near the feeding site (2 cm length) was used for staining. Each stem was cut into slices horizontally of 101 μm thickness and then stained in 1% (w/v) phloroglucinol liquor for 5 min. Slices were then mounted on slides and a few drop of HCl (18%, v/v) were added. The extent of staining was examined by a Leica microscope.

### Determination the Activity of 4-Coumarate: CoA Ligase and Cinnamyl Alcohol Dehydrogenase

The activity of 4-Coumarate: CoA Ligase (4CL) was examined by the increase in A_333_ with *p*-coumarate as substrate. The reaction mixture contained crude enzyme, 0.2 mM *p*-coumarate, 0.8 mM ATP, 7.5 mM magnesium chloride anhydrous, and 38 μM CoA in 100 mM TES (pH 7.6). Protein concentrations were determined by the approach of Bradford (1976). 4CL activity was expressed as 0.01 ΔOD333/mg protein (Knobloch and Hahlbrock, 1977; Shan et al., 2008).

For the determination of CAD activity, the crude enzyme was incubated with 2 mM NADP and 1 mM *trans-*cinnamic acid at 37°C for 30 min and then terminated by adding 1 M HCl. One unit of CAD was defined as the amount of enzyme that caused a change of 0.01 in absorbance per milligram protein at 37°C (Liu et al., 2014).

### Quantitative Real-Time PCR Analysis

Differential expression of selected genes was verified by quantitative real-time PCR (qRT-PCR) (Liu et al., 2016). Total RNA from the treated stem and leaves was extracted with Eastep^TM^ Total RNA Extraction Kit (Progema). First-strand cDNA was synthesized from 1 μg of RNA using GoScript^TM^ Reverse Transcription system (Promega). qPCR amplification was carried out using *OsActin* as endogenous control. SYBR Green probes for each gene were used. The primers are listed in Supplementary Tables 1, 2. Real-time PCRs proceeded with 5 μl of the 2 × UltraSYBR Mixture, 0.2 μl (0.2 μM) of each specific primer, and 1 μl of cDNA, and the final volume was adjusted to 10 μl with RNase-free water. The thermal cycle reaction condition was as follows: initial denaturation at 95°C for 10 min, 40 cycles of 95°C for 15 s and 60–64°C for 1 min. The specificity of amplicons was verified by the melting curve analysis. Three independent biological replicates for each treatment were used for qRT-PCR analyses.

### Metabolome Analysis

Rice seedlings were treated with different nitrate levels for 30 days and then infected with SSB for 3 days. The feeding stem (3 cm from feeding sites) was collected and then stored at −80°C. For LC-MS analysis, frozen stem was ground in liquid N_2_ and lyophilized overnight. A mixture of methanol and H_2_O (70:30, 1.2 mL) was added to 100 mg of lyophilized tissue, shaken per 2–3 h overnight, and centrifuged for 10 min at 12,000 *g*. Aliquots of supernatant were transferred to clean tubes; the clear supernatant was filtered into glass LC autosampler vials using a PVDF filter and stored at −20°C until analyzed. Samples were analyzed by UPLC-MS/MS (ultra-performance liquid chromatography–tandem mass spectrometry). Extraction for UPLC-MS analysis was adapted from the study by Fraga et al. (2010).

### RNA-Seq

Plants cultured for 30 days were infested with third instars of SSB for 24 h and then sampled for total RNA extraction with mirVana miRNA isolation kit (Ambion). The required fragments were sequenced using Illumina HiSeq 2500 instrument, with default quality parameters, at Oebiotech (Shanghai, China). The reference genome was obtained according to the previous study (Kim et al., 2015). Both portals were included in this study to allow a complete analysis of the genome. NGS QC Toolkit was used for quality control of the raw reads and then aligned to the reference genome (Patel and Jain, 2012). The software R package of DESeq was employed to capture the differentially expressed genes (DEGs) (Anders and Huber, 2012). All unigenes were annotated with gene ontology (GO) and KEGG pathway analysis.

### Data Analysis

SPSS 22.0 (SPSS, Chicago, IL, United States) was used for statistical analysis. Data were assessed by three-way ANOVA, two-way ANOVA, one-way ANOVA, and independent-sample *t*-test, using Tukey’s test for differences between means.

## Results

### Low Nitrate Supply Enhances Rice Anti-herbivore Defense

**FIGURE 1:**
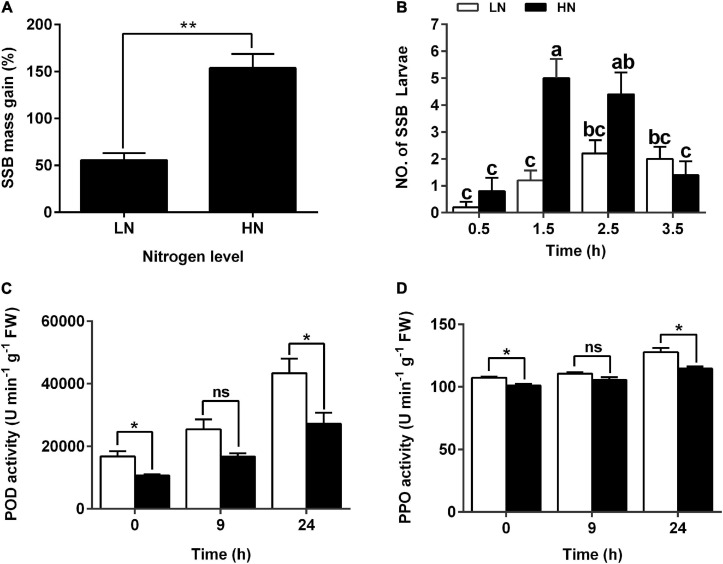
Larval performance of the striped stem borer (SSB) and defensive enzyme activities in rice plants cultivated with different concentrations (0.3 and 3 mM) of nitrate. **(A)** Mass gain of SSB larvae fed on rice plants cultured with different concentrations of nitrogen. Seven-day-old seedlings were transplanted to nutrient solution containing 0.3 mM KNO_3_ (LN) or 3 mM KNO_3_ (HN) and cultured for 30 days. In low nitrogen treatment, potassium chloride was used to replenish potassium. Larvae at the third-instar stage were used for bioassays. The individual larvae were measured 3 days after inoculation, and the mean percentage of mass gain was calculated. Values are mean ± SE (*n* = 20). Asterisks (**) indicate Student’s *t*-test significance at *P* < 0.01 versus the indicated samples. **(B)** Feeding preference of SSB on rice plants cultured under nitrogen supply. The number of SSB larvae fed on rice plants was recorded at 0.5, 1.5, 2.5, and 3.5 h after SSB inoculation. Each data is the mean ± SE of five replicates. Different letters indicate statistically significant differences between treatments (Tukey’s multiple range test: *P* < 0.05). Enzyme activity of POD **(C)** and PPO **(D)** in the stems of rice plants cultivated with different concentrations (0.3 and 3 mM) of nitrate and inoculated with the striped stem borer (SSB). Values are mean ± SE of three replicates. Asterisks (*) indicate Student’s *t*-test significance at *P* < 0.05 versus the indicated samples, and ns means no significant difference.

To investigate the effects of nitrogen levels on rice defense against herbivore, rice seedlings were cultured with modified Kimura B nutrient solution containing different concentration of KNO_3_ (0.3 mM for LN and 3 mM for HN) for 30 days and then infested with third instars of SSB for 3 days. Long LN treatment decreased seedling height and aboveground dry weight, while it increased the root length and underground dry weight, compared with HN treatment (Supplementary Figure 1). Additionally, 55.54 and 153.8% increases in SSB mass gain were found in the larvae fed on plants cultured with LN and HN, respectively (Figure 1A and Supplementary Figure 2). It seems that LN could enhance rice defense against SSB infestation. The feeding preference of SSB larvae was significantly different between rice plants cultured under LN and HN supply 1.5 h after larval inoculation. SSB preferred to HN-cultured plants (Figure 1B). Moreover, the plants cultured with LN exhibited an enhanced activity of PPO and POD compared with those with HN (Figures 1C,D). Collectively, the nitrate supply could regulate seedling anti-herbivore defense, and LN enhanced plant defense compared with that in HN treatment.

### Nitrate Supply Alters Rice Primary Metabolism and Chlorophyll Metabolism Under SSB Infestation

**FIGURE 2:**
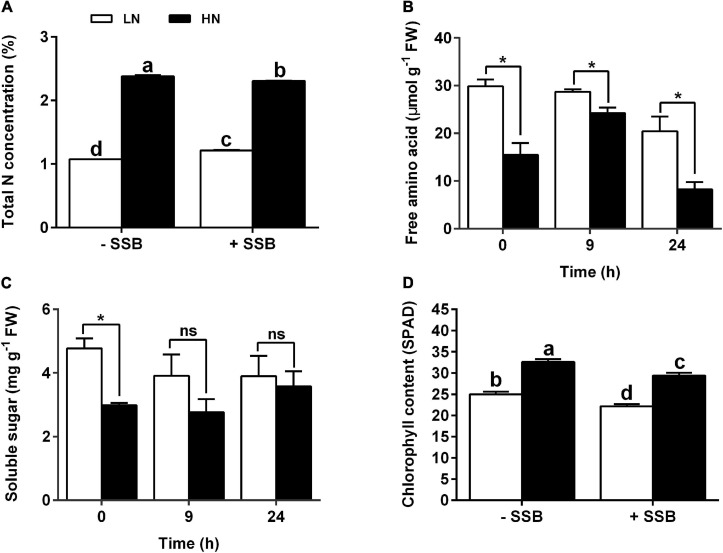
Changes in nitrogen contents, primary metabolite, and chlorophyll metabolism in rice plants cultivated with different concentrations (0.3 and 3 mM) of nitrate and inoculated with the striped stem borer (SSB). Seven-day-old seedlings were transplanted to nutrient solution containing 0.3 mM (LN) or 3 mM KNO_3_ (HN) and cultured for 30 days. **(A)** Total nitrogen content was determined at 48 h after SSB inoculation. The contents of soluble sugar content **(B)** and free amino acid content **(C)** were determined at 0, 9, and 24 h after SSB inoculation. **(D)** Total chlorophyll content was determined at 24 h after SSB inoculation. Data are the mean ± SE of three replicates **(A–C)** and 12 replicates **(D)**. Different letters indicate statistically significant differences between treatments (Tukey’s multiple range test: *P* < 0.05). Asterisks (*) indicate Student’s *t*-test significance at *P* < 0.05 versus the indicated samples, and ns means no significant difference.

To investigate the effect of nitrate supply on metabolism, total nitrogen content, total FAA content, and soluble sugar content were determined. To be consistent with the treated nitrogen concentration, the plants cultured with HN exhibited higher total nitrogen content LN regardless of SSB inoculation or not (Figure 2A). In the first 24 h of infestation, free amino acid content was decreased. Intriguingly, the content of free amino acid in plants cultured with LN was higher than that with HN independent of SSB inoculation (Figure 2B). However, no significant difference in soluble sugar was found between HN and LN treatments under SSB infestation (Figure 2C). Moreover, chlorophyll content was decreased after SSB inoculation, and chlorophyll content in the plants cultured with HN was significantly higher than that in LN (Figure 2D). qPCR analysis showed that the expression of gene (*NYC1*, *PAO*) involved in chlorophyll degradation was upregulated under SSB infestation, and the effect was more obvious in LN than that in HN (Supplementary Figures 3A,C and Supplementary Table 1). The results show that nitrate supply levels alter primary metabolism and chlorophyll metabolism in SSB-infested rice plants.

### Nitrate Supply Alters the Primary and Secondary Metabolism, as Well as Constitutive Defense

**FIGURE 3:**
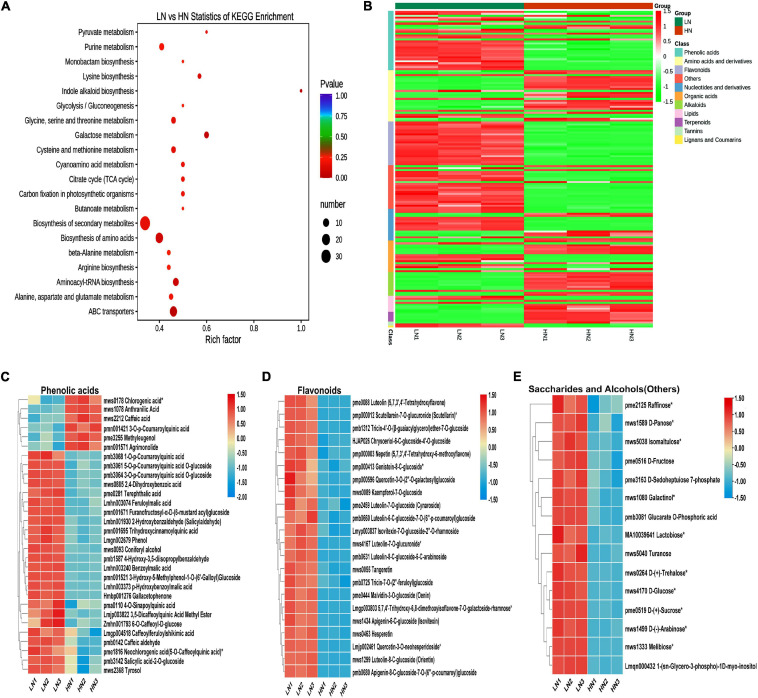
Metabolome profiling of the stems of rice plants cultivated with different concentrations (0.3 and 3 mM) of nitrate and inoculated with the striped stem borer (SSB). Seven-day-old seedlings were transplanted to nutrient solution containing 0.3 mM (LN) or 3 mM KNO_3_ (HN) and cultured for 30 days for sampling for metabolome analysis. **(A)** KEGG enrichment analysis of difference in metabolites between LN and HN treatments. Heatmap analysis of differentially accumulated metabolites **(B)**, phenolic acids **(C)**, flavonoids **(D)**, and saccharides and alcohols **(E)** between LN and HN treatments. In order to facilitate comparison, the relative content of metabolites was normalized.

Metabolome analysis was conducted to investigate the effect of nitrate supply on rice cell metabolism. The quality control for metabolome was shown as PCA (Supplementary Figure 4). KEGG pathway enrichment analysis was used to study the signaling pathways of all detectable metabolites (Kanehisa et al., 2008). It was shown that differentially accumulated compounds (DACs) were significantly enriched in the biosynthesis of secondary metabolites (Figure 3A). Further analysis showed that low nitrate significantly increased the production of phenolic acids, flavonoids, saccharides, and alcohols (others), while high nitrate significantly increased the accumulation of amino acids and their derivatives, alkaloids and terpenoids (Figure 3B). Each metabolite group of the phenolic acids, flavonoids, saccharides, and alcohols is shown in Figures 3C–E, respectively.

To further evaluate the effects of the transcriptome change on the metabolome, RNA-Seq was taken. The RNA-Seq metrics for quality control was shown as PCA, correlation analysis of differential genes (Supplementary Figure 5). Additionally, the transcriptomes were also validated through quantitative PCR (qPCR) analysis of selected genes over the treatment, including β-actin as an endogenous control (Supplementary Table 2). The results of RNA-Seq were consistent with those of qPCR. The overview of differential genes under nitrogen treatment is shown in Supplementary Figure 5C (*P* < 0.05, fold change > 2, FPKM ≥ 5), 1,217 DEGs were found in data between LN and HN treatments, while 638 DEGs were between LNSBB and HNSSB treatments. About 164 DEGs were co-expressed between LN and HN, and LNSSB and HNSSB (Supplementary Figure 5D).

**FIGURE 4:**
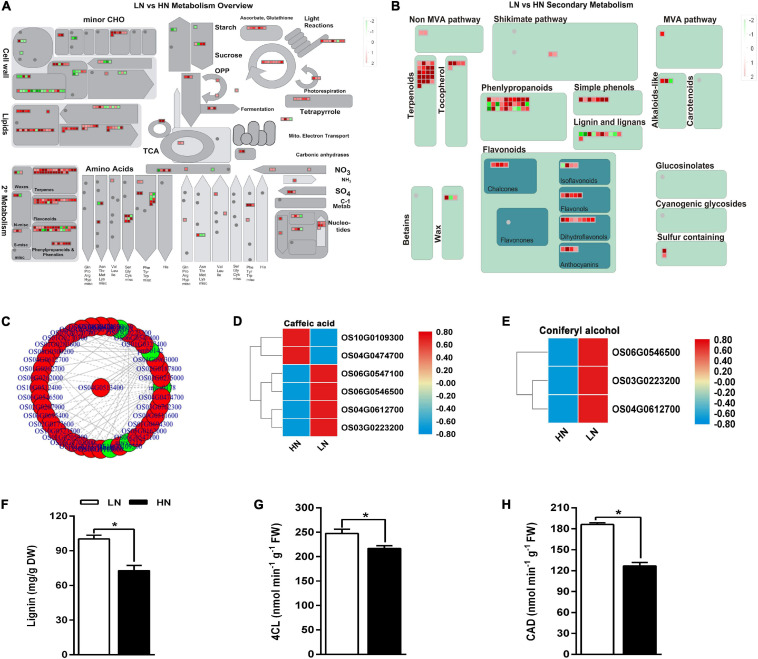
Joint analysis of transcriptome and metabolome of the stems of rice plants cultivated with different concentrations (0.3 and 3 mM) of nitrate and inoculated with the striped stem borer (SSB). Seven-day-old seedlings were transplanted to nutrient solution containing 0.3 mM (LN) or 3 mM KNO_3_ (HN) and cultured for 30 days for sampling. The overview of differential metabolites **(A)** and differential secondary metabolites **(B)**. Red means HN upregulated, while green means HN downregulated. **(C)** Joint analysis of the correlation network associated with phenylpropanoids pathway. Relative expression levels of genes involved in the biosynthesis of caffeic acid **(D)** and coniferyl alcohol **(E)**. Quantitative evaluation of lignin content **(F)** and activity of 4CL **(G)** and CAD **(H)**. Asterisks (*) indicate Student’s *t*-test significance at *P* < 0.05. To facilitate comparison, normalize gene expression levels. 4CL, 4-Coumarate: CoA ligase, CAD, Cinnamyl alcohol dehydrogenase.

The DEGs and DACs were incorporated in the KEGG pathway (Figure 4A). Additionally, the effects of transcriptome change on the secondary metabolites were also evaluated (Figure 4B). The great changes occurred in cell wall, sugar, and lignin pathway in LN. Genome-wide connection network between phenylpropanoid metabolism-associated genes and metabolites was analyzed in Figure 4C. Caffeic acid and coniferyl alcohol are two important intermediate metabolites of lignin biosynthesis (Zhao and Dixon, 2011). Four of six genes associated with caffeic acid metabolism and all three genes associated with coniferyl alcohol metabolism were upregulated under low nitrate regime (Figures 4D,E). The activity of 4CL and CAD, which are involved in the lignin biosynthesis, were upregulated under low nitrate supply (Figures 4G,H), resulting in the accumulation of lignin (Figure 4F). These results indicated that low nitrate supply might regulate rice defense against insect herbivory through lignin deposition.

### Metabolism Alteration Under SSB Infestation Induced by Nitrate Supply

**FIGURE 5:**
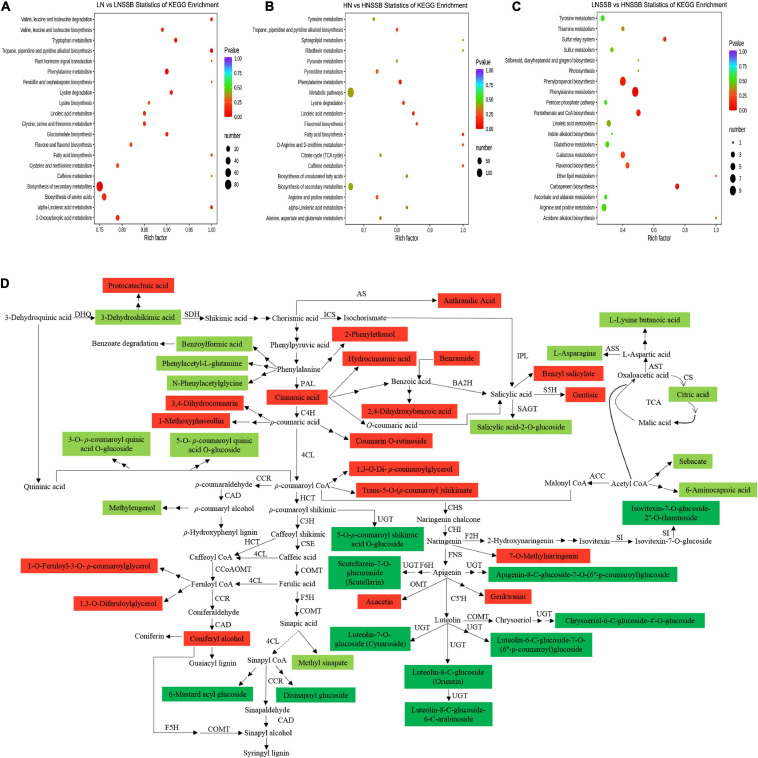
Overview of metabolism changes in the stems of rice plantscultivated with different concentrations of nitrate and inoculatedwith the striped stem borer (SSB). Seven-day-old seedlings were transplanted to nutrient solution containing 0.3 mM (LN) or 3 mM KNO_3_ (HN) and cultured for 30 days for sampling. KEGG enrichment analysis of differential metabolites in rice between LN and LNSSB treatments **(A)**, HN and HNSSB treatments **(B)**, and LNSSB and HNSSB treatments **(C)**. **(D)** Alteration of metabolites involved in shikimic acid and TCA pathways under nitrogen and SSB treatment. Red means that the metabolites were upregulated after SSB infestation both in LN and in HN treatment and the metabolites in LNSSB group were less than those in HNSSB group. Light green means that the metabolites were upregulated after SSB infestation both in LN and in HN treatment and the metabolites in LNSSB group were more than those in HNSSB group. Dark green means that the metabolites were downregulated after SSB infestation both in LN and in HN treatment but the metabolites in LNSSB group were more than those in HNSSB group. LNSSB, 0.3 mM KNO_3_ cultured for 30 days, then infected by SSB for 3 days; HNSSB, 3 mM KNO_3_ cultured for 30 days, then infected by SSB for 3 days. Single solid arrow means the established biosynthesis steps, two solid arrows means the involvement of multiple enzymatic reactions, while broken arrows represent the unestablished biosynthesis steps. DHQ, 3-dehydroquinate dehydratase; SDH, shikimate dehydrogenase; AS, anthranilate synthase; ICS, isochorismate synthase; IPL, isochorismate pyruvate lyase; BA2H, benzoic acid 2-hydroxylase; SAGT, salicylic acid glucosyltransferase; S5H, salicylate 5-hydroxylase; AST, aspartate aminotransferase; ASS, asparagine synthase; CS, citrate synthase; ACC, acetyl-CoA carboxylase; PAL, phenylalanine ammonia lyase; C4H, cinnamate 4-hydroxylase; 4CL, 4-coumarate: CoA ligase; CCR, cinnamoyl-CoA reductase; HCT, hydroxycinnamoyl-CoA: Shikimate/quinate hydroxycinnamoyl-transferase; C3H, ρ-coumarate 3-hydroxylase; CCoAOMT, caffeoyl-CoA *O*-methyltransferase; F5H, ferulate 5-hydroxylase; CSE, caffeoyl shikimate esterase; COMT, caffeic acid *O*-methyltransferase; CAD, cinnamyl alcohol dehydrogenase; UGT, UDP-glycosyltransferase; CHS, chalcone synthase; CHI, chalcone isomerase; FNS, flavonoid synthase; F6H, flavanone-6-hydroxylase; OMT, *O*-methyltransferase; C5′H, dihydroflavonol-5′-hydroxylase; F2H, flavanone-2-hydroxylase; SI, isovitexin beta-glucosyltransferase.

For the combination of nitrogen and SSB treatment, the metabolites in rice plants were divided into 12 classes according to the tendency of changes (Supplementary Figure 6). KEGG pathway-enrichment analysis was also used to determine the involved signaling pathways of the DACs under SSB infestation. Most of DACs were significantly enriched in the biosynthesis of secondary metabolites and metabolic pathways under LN (Figure 5A) and HN (Figure 5B) supply. Moreover, the first two enriched pathways were phenylalanine metabolism and phenylpropanoid biosynthesis in HNSSB compared with LNSSB (Figure 5C).

To examine the effects of nitrogen supply on primary and secondary metabolism in induced defense, the DACs were further divided into three classes (Figure 5D): (1) The metabolites were upregulated after SSB infestation both in LNSSB and in HNSSB group and the metabolites in LNSSB group were less than those in HNSSB group (red marked in Figure 5D); (2) the metabolites were upregulated after SSB infestation both in LNSSB and in HNSSB group and the metabolites in LNSSB group were more than those in HNSSB group (light green marked in Figure 5D); (3) the metabolites were downregulated after SSB infestation both in LNSSB and in HNSSB group but the metabolites in LNSSB group were more than those in HNSSB group (dark green marked in Figure 5D).

**FIGURE 6:**
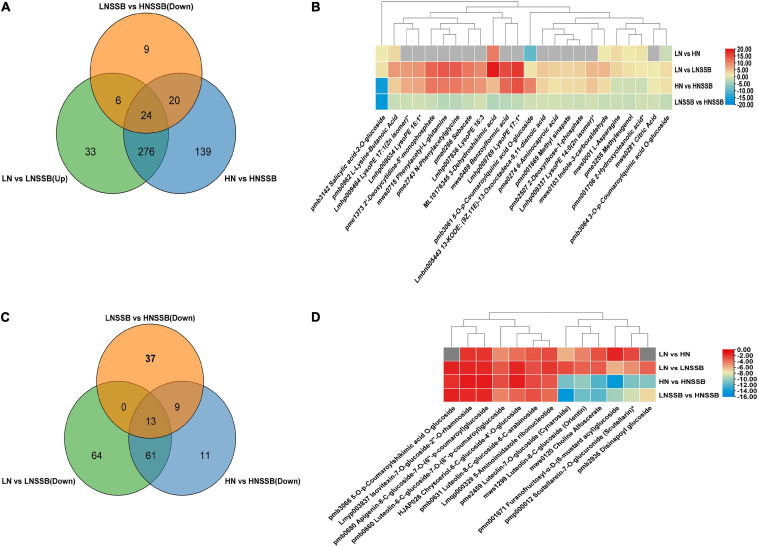
Metabolites involved in rice responses to nitrogen deficiency and insect herbivory by the striped stem borer (SSB). LN, HN, and LNSSB are the control in the group LN vs. LNSSB, HN vs. HNSSB, and LNSSB vs. HNSSB, respectively. **(A)** Venn diagram analysis of differential metabolites under treatment from induced defense, which was upregulated after SSB infestation both in LN and in HN treatment, and the content of that in LNSSB group is more than that in HNSSB group. **(B)** Heatmap analysis of differential metabolites shared under treatment from induced defense (24). **(C)** Venn diagram analysis of differential metabolites under treatment from anti-defense, which was downregulated after SSB infestation both in LN and in HN treatment, and the content of that in LNSSB group is more than that in HNSSB group. **(D)** Heatmap analysis of differential metabolites shared under treatment from anti-defense (13). Gray square indicates no significant differences between metabolites. LN, 0.3 mM KNO_3_ cultured for 30 days; HN, 3 mM KNO_3_ cultured for 30 days; LNSSB, 0.3 mM KNO_3_ treatment for 30 days, then infected by SSB for 3 days; HNSSB, 3 mM KNO_3_ treatment for 30 days, then infected by SSB for 3 days.

The source of red marked compounds were approximately divided into five classes: cinnamic acid, *p*-coumaric acid, *p*-coumaric CoA, feruloyl CoA and apigenin. The light green marked compounds were screened by Venn diagram analysis (Figure 6A). And they were almost derived from four compounds: 3-dehydroquinic acid, phenylalanine, acetyl CoA, and aspartic acid. Each content of the compounds is shown in Figure 6B. The dark green marked compounds were derived from about three compounds: sinapyl CoA, apigenin, and luteolin. These compounds were screened (Figure 6C), and the content of the compounds are shown in heatmap (Figure 6D).

**FIGURE 7:**
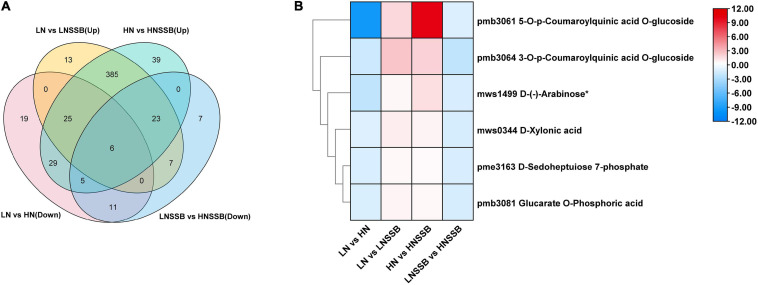
Priming metabolites possibly involved in defense under low nitrogen and insect herbivory by the striped stem borer (SSB). LN, LN, HN, and LNSSB are the control in the group LN vs. HN, LN vs. LNSSB, HN vs. HNSSB, and LNSSB vs. HNSSB, respectively. **(A)** Venn diagram analysis of differential metabolites under treatment, which was the content of that in LN group more than that in HN group before SSB infestation, and the content of that in LNSSB group is more than that in HNSSB group after SSB infestation. **(B)** Heatmap analysis of differential metabolites shared under treatment (six). LN, 0.3 mM KNO3 cultured for 30 days; HN, 3 mM KNO3 cultured for 30 days; LNSSB, 0.3 mM KNO3 cultured for 30 days, then infected by SSB for 3 days; HNSSB, 3 mM KNO3 cultured for 30 days, then infected by SSB for 3 days.

Additionally, Venn diagram analysis showed that six metabolites were induced by both nitrate and SSB, and the content of induced metabolites in LNSSB group was more than that in HNSSB group (Figure 7A). These metabolites were 3-*O*-**p**-coumaroylquinic acid *O*-glucoside, 5-*O*-**p**-coumaroylquinic acid *O*-glucoside, D-xylonic acid, D-(-)-arabinose, D-sedoheptulose 7-phosphate, and glucarate *O*-phosphoric acid (Figure 7B).

### Alteration of Lignin Content Under Nitrate and SSB Treatment

Before SSB inoculation, the lignin content in LN-cultured plants was higher than that in HN-cultured plants (Figure 4F). The vast majority of compounds involved in lignin biosynthesis were upregulated by SSB feeding (Supplementary Figure 7A). Interestingly, after SSB infestation, lignin accumulation was higher (Supplementary Figure 7B) and faster in HN-cultured plants (Supplementary Figure 7C).

### Effect of Nitrate Supply on Phytohormone Level Under SSB Infestation

**FIGURE 8:**
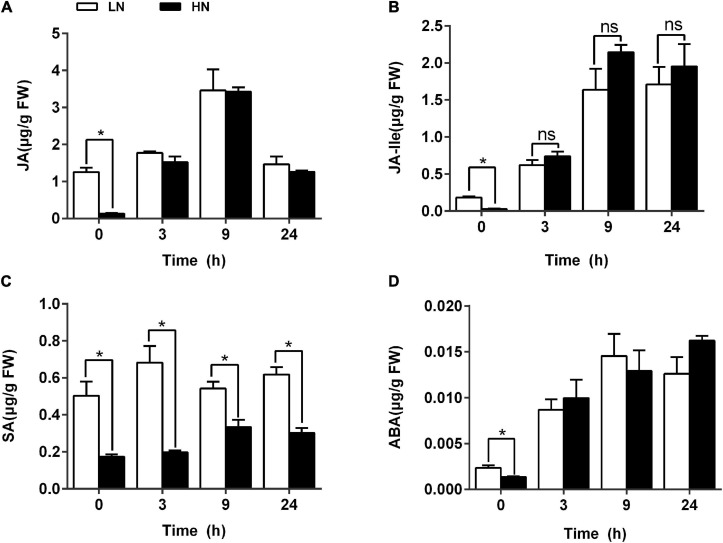
Levels of phytohormones in rice plants cultivated with different concentrations (0.3 and 3 mM) of nitrate and inoculated with the striped stem borer (SSB). Seven-day-old seedlings were transplanted to nutrient solution containing 0.3 mM KNO_3_ or 3 mM KNO_3_ for 30 days. The contents of JA **(A)**, JA-Ile **(B)**, SA **(C)**, and ABA **(D)** were determined by LC-MS at 0, 3, 9, and 24 h after SSB inoculation under nitrogen supply. Each bar is the mean ± SE of three replicates. Asterisks (*) indicate Student’s *t*-test significant at *P* < 0.05 versus the indicated samples, and ns means no significant difference.

To determine the effects of nitrogen levels on phytohormones possibly involved in plant defense against herbivores, LC-MS analysis was performed to quantify phytohormone levels of JA, JA-Ile, SA, and ABA in rice plants cultivated with different concentrations (0.3 and 3 mM) of nitrate and inoculated with the SSB. Phytohormone standard curve is shown in Supplementary Figure 8. Higher contents of JA and JA-Ile were found in LN-cultured plants before SSB inoculation. However, no significant difference in JA and JA-Ile contents was detected between LN- and HN-cultured plants 3 h after SSB inoculation (Figures 8A,B). However, constantly higher content of SA was found in LN-cultured plants either before or after SSB inoculation (Figure 8C). The changes in ABA contents were similar to those in JA and JA-Ile (Figure 8D). To further determine the role of JA signaling in rice defense against SSB infestation under different regimes of nitrogen, two rice RNAi lines *aos* RNAi and *coil* RNAi of the JA signaling pathway were used. The two transgenic lines were obtained by silencing the expression of allene oxide synthase (*OsAOS*; active in JA biosynthesis) and CORONATINE INSENSITIVE1 (*OsCOI1*; active in JA perception) genes in rice plants *via* RNAi (Ye et al., 2012). Silencing either *OsAOS* or *OsCOI1* enhanced rice susceptibility to SSB infestation. Increased nitrogen supply decreased rice resistance to SSB regardless of the genotypes (Supplementary Figure 9), suggesting independence of JA signaling in nitrogen-mediated anti-herbivore defense in rice plants.

## Discussion

Nutrient status plays a key role in plant defense against insect herbivores. Fertilization management may serve as an important approach to manage insect pests in sustainable agriculture. In this study, low nitrogen treatment for 4 weeks significantly enhanced rice defense against SSB (Figure 1), one of the most destructive rice pests. Nitrogen deficiency changed both primary and secondary metabolism of rice, especially phenylpropanoid metabolism (Figures 2–4), leading to differential metabolic bypass in rice defense against SSB herbivory.

### Trade-Off Between Growth and Defense Induced by Plant Nutrition

In plants, trade-offs exist between growth and resistance to herbivory because secondary metabolism and physical defenses divert resources from plant growth (Herms and Mattson, 1992; Huot et al., 2014; Züst and Agrawal, 2017). Immune-triggered diminished growth is a strategy to avoid starvation of essential metabolic intermediates, which is generally consistent with the acclimatory response hypothesis, i.e., diminished growth may optimize the temporal and spatial expression of defense compounds without compromising other critical roles in central metabolism (Guo et al., 2018).

Previous studies have revealed that nutrient availability could regulate plant trade-offs between growth and defense. Increased nitrogen availability decreased plant resistance to the three herbivores in cranberry, regardless of genotypes (de Lange et al., 2019). Nitrogen fertilizer affects ecological fitness of herbivores, such as selection to host plants, survival, growth, development, fecundity, and population dynamics (Lu et al., 2007). Nutrient availability can influence plant resistance to herbivores in various ways, such as by altering plant quality as a food source or by changing levels of secondary metabolites (Bryant et al., 1987; Altieri and Nicholls, 2003). Increasing nutrient availability may alter plant carbon allocation to chemical defensive compounds (Guo et al., 2018). Meanwhile, it also modify carbon allocation to structural defensive compounds to achieve their physical defense strategies in response to nutrient enrichment (Scalbert, 1991; Close and McArthur, 2002).

### Metabolites Adjustment Induced by Nitrate in Rice Anti-herbivore Defense

Metabolome change is a strategy used by plants to cope with changes in the external environment. However, there are few related reports on priming mechanisms at the metabolome level.

Under SSB infestation, the content of chlorophyll was decreased independent of nitrogen supply (Figure 2D), which might be resulted in the reduced photosynthetic activity. Therefore, plants could face increased energetic and carbon demands to support inducible defenses. In comparison with rice with replete nitrate, nitrogen deficiency induced the accumulation of saccharides in the stem before SSB infection (Figure 3E). Once rice was infected by SSB, nitrogen deficiency promotes the degradation of saccharides; it is just the opposite for HN treatment. Therefore, rice cultured with nitrate deficiency could provide more energy from local catabolism of saccharides. Besides, the accumulation of pentoses (such as melibiose) in nitrate-deficient rice stem was also linked to the production of phenolic molecules (Gardiner, 1966).

In response to stress environments, such as drought, plants produce more phenolic compounds to serve as enzymatic antioxidants to scavenge excess ROS (Hatier and Gould, 2008; Agati and Tattini, 2010). Additionally, phenolic acid is also a leading indicator of grain resistance or susceptibility to insects (Classen et al., 1990). Previous studies have been revealed that nitrogen fertilization decreased the levels of phenolic compounds in corn (Ren et al., 2013) and tomato (Stout et al., 1998), as well as the levels of other defensive compounds in tomato (Hoffland et al., 2000; Larbat et al., 2016), peach (*Prunus persica*) (Sauge et al., 2010), and cotton (Chen et al., 2008). In this study, we also found that nitrate deficiency induced the accumulation of phenolic acids and flavonoids (Figures 3C,D), which might be resulted in the increase of constitutive defense.

The former study has been revealed that nitrogen deficiency leads to a marked shift from the nitrogen-containing alkaloid nicotine to carbon-rich phenylpropanoids (Fritz et al., 2006). The analysis of metabolome under nitrogen supply was also shown that the phenylpropanoid pathway was significantly different between HN group and LN group under SSB infestation (Figure 5C). The phenylpropanoid pathway is essential in plants, providing precursors for numerous secondary metabolites, including monolignols, flavonoids, and coumarins (Fraser and Chapple, 2011). The stimulation of phenylpropanoid metabolism is triggered by changes of nitrogen, which is mediated by the induction of a set of enzymes in the early steps of the phenylpropanoid biosynthetic pathway (such as 4CL). The differential nitrogen supply also leads to the flux of carbon into phenylpropanoids metabolism in different route under SSB infestation (Figure 5D).

Nitrogen deficiency promoted the accumulation of specific organic acids, phenolic acids, and saccharides before SSB infestation (Figures 3B,C,E). Organic acids are a unique group of metabolites, which are intermediate metabolites of critical metabolic pathways, such as the Krebs cycle, carbohydrate metabolism, ketone body metabolism, fatty acid β-oxidation, neurotransmitters turnover, and protein metabolism (Tsoukalas et al., 2017). Saccharides are omnipresent and important components for general metabolism. Recent studies show that sugars can act as critical signaling molecules in regulation of cellular metabolism in response to biotic and abiotic stress (Sheen et al., 1999; Smeekens and Hellmann, 2014). Interestingly, organic acids (such as D-xylonic acid), phenolic acids (such as 3-*O*-**p**-coumaroylquinic acid *O*-glucoside and 5-*O*-**p**-coumaroylquinic acid *O*-glucoside), and saccharides [such as D-(-)-arabinose, D-sedoheptulose 7-phosphate, and glucarate *O*-phosphoric acid] might have a similar priming mechanism effect under SSB infestation (Figure 7). Priming is operative through a complex network of signaling pathways. The advantage of priming is that it offers the plant an enhanced protection without the costs of constitutively expressing their defense genes.

### The Differential Regulation of Lignin Under Nitrogen Supply in Constitutive and Inducible Defense

Lignin is important for terrestrial plants by providing a structural support for the upward growth of plants and enabling the long-distance water transportation (Humphreys and Chapple, 2002). However, increased lignin accumulation is also harmful for plant growth. In plants, there are two major steps to produce lignin: monolignol biosynthesis and monolignol polymerization *via* free radical coupling. Previous study has been revealed that the levels of nitrate supplied in solution influenced the lignin production (Fritz et al., 2006; Comadira et al., 2015). In our system, we also found that low nitrate levels resulted in an increase in lignin in rice stem (Figure 4F). High content of lignin could make plants less palatable to herbivores, which can decrease litter decomposability and the rates of nutrient cycling concomitantly (Ostrander and Coors, 1997; Hideki and Takayuki, 2011). Additionally, secondary cell walls consisted of lignin also play a key role as a passive barrier in the defense against SSB infestation. Therefore, the higher accumulation of lignin in nitrate-deficient rice could promote the constitutive defense.

It is worth noting that the content of lignin in nitrate-replete rice was more than that in nitrate-deficient rice under SSB infestation for 3 days (Supplementary Figure 7). Plants cells possess various types of sensors at the plasma membrane (and possibly in the cell wall) that can probe mechanical deformations or changes in cell wall structure or composition by a rapid growth inhibition coupled with the production of ROS, ACC, and jasmonate (Wolf et al., 2012). The more injured cell wall in nitrate-replete rice might induce more deposition of lignin to reinforce their cell walls.

### Phytohormone in Low-Nitrogen-Induced Priming Against SSB Infestation

Jasmonic acid is one of the most important hormones involved in the response of plants to herbivory-induced wounding, controlling the majority of insect-regulated genes in Arabidopsis leaves (Acosta and Farmer, 2010). The level of plant nutrition was closely related to the ability of rice anti-herbivore defense at least partially by regulating phytohormone signal. Previous study had revealed that Pi deficiency induced JA pathway and triggered increased resistance to *Spodoptera littoralis* in Arabidopsis, tomato, and *Nicotiana benthamiana* (Khan et al., 2016). Here, it was revealed that JA content was higher in LN group than that in HN group before SSB infection (Figure 8A). Previous study revealed that JA signal could regulate the production of volatile compounds, resulting in the difference in insect selectivity (Paré and Tumlinson, 1999), which was consistent with our results that the number of feeding SSB in LN group was more than that in HN group in the initial feeding time analyzed by feeding preference (Figure 1B). However, whether the regulation of JA signal on insect selectivity was affected by nitrate supply should be investigated in the future.

Additionally, we also found that there was no significant difference between LN and HN group after 3-h infection with SSB (Figure 8A). And the knockdown of JA signal did not significantly change the feeding of SSB under nitrate supply (Supplementary Figure 9). It seems that nitrate deficiency is not the same as Pi deficiency in rice defense against chewing herbivore infestation. Previous study has been revealed the complex signaling networks arising from cell wall alterations and leading to the upregulation of JA biosynthesis (Mielke and Gasperini, 2019). MeJA treatment prevents isoxaben-induced lignification in Arabidopsis in a concentration-dependent manner (Denness et al., 2011). Meanwhile, cell wall-degrading enzymes and cell wall fragments play a major role as triggers of the JA pathway (Ellis et al., 2002; Bömer et al., 2018). The accumulation of lignin in LN group might inhibit the initialization of JA signal in the process of rice defense against SSB infestation. Additionally, it was also found that SA levels in LN group were always higher than those in HN group in 1-day infestation by SSB (Figure 8C). Generally, JA signal was antagonized by SA signal in rice defense against chewing herbivore infestation, which also might lead to the similar levels of JA content in LN and HN groups after 3-h inoculation by SSB.

Besides, other phytohormones might also be involved in rice defense against SSB infestation under nitrogen supply. Previous study has revealed that LN could induce the accumulation of auxin in plant (Krouk, 2016; Sun X. et al., 2020). And herbivory-induced auxin promotes the production of anthocyanins and phenolamides in *Nicotiana attenuate* (Machado et al., 2016). Additionally, recent research has also revealed that auxin and ABA signals have a synergistic effect in plant response to drought stress. It seems that auxin could also play a positive role in stress response (Zhang et al., 2012; Wang et al., 2019). Auxin, ethylene (ET), and ABA are stress-related phytohormones that are induced upon herbivory and are well-established modulators of plant resistance to herbivores. Unfortunately, ABA content in LN group was no significantly different from that in HN group under SSB infestation (Figure 8D). However, previous study has revealed that auxin could promote the transduction of ET signal (Fei et al., 2017; Yue et al., 2020). Therefore, the relationship between auxin and ET signals in rice defense against SSB herbivory should be investigated in the future.

### Strategy Adjustment of Nitrogen Application-Cost Less but Defense More

The expression of fitness costs depends on environmental conditions such as nutrient availability. Slow-growing plant species, which typically evolved in resource-limited environments, are less able to replace the lost tissue than fast-growing plant species from more competitive environments and should therefore invest in constitutive rather than in induced defense (Kempel et al., 2011). In this study, we found that nitrate deficiency promotes the accumulation of specific organic acids, phenolic acids, saccharides, and lignin, which might be involved in priming of rice defense against SSB infestation. Generally, the benefits of priming outweigh its costs when stress occurs. Therefore, priming is a fine economic solution to the trade-off dilemma between plant defense protection and costs involved in enhancing defense responses (Conrath et al., 2006). The large amount of energy invested in lignin and its precursors has the potential to compensate the costly expenditure of defense, which consequently would mitigate the trade-off between growth and defense.

**FIGURE 9:**
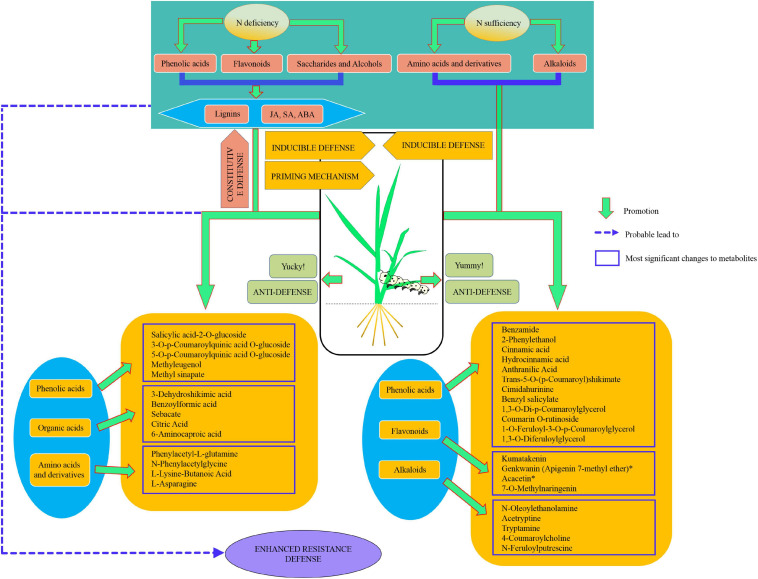
Model for metabolome responses to nitrogen deficiency and insect herbivory by the striped stem borer (SSB) in rice plants.

In conclusion, our results showed that nitrogen deficiency enhanced rice resistance to SSB. Nitrogen deficiency and sufficiency motivated the accumulation of different metabolites outlined in Figure 9. Nitrogen deficiency promoted the accumulation of phenolic acids, flavonoids, saccharides, and alcohols and, in particular, promoted the accumulation of lignin, while nitrogen sufficiency promoted the accumulation of amino acids and derivatives, as well as N-contained alkaloids. Upon insect herbivory, nitrogen deficiency may tend to initiate plant constitutive defense by the accumulation of phenolic acids and flavonoids.

## Data Availability Statement

The data presented in the study are deposited in SRA database, accession number (PRJNA742516).

## Author Contributions

RZ, JL, and YS conceived and designed the experiments. YZ performed the experiments and analyzed the data. XZ, XL, NQ, and KX analyzed the data. YZ, RZ, and JL wrote and revised the manuscript. All authors read and approved the final manuscript.

## Conflict of Interest

The authors declare that the research was conducted in the absence of any commercial or financial relationships that could be construed as a potential conflict of interest.

## Publisher’s Note

All claims expressed in this article are solely those of the authors and do not necessarily represent those of their affiliated organizations, or those of the publisher, the editors and the reviewers. Any product that may be evaluated in this article, or claim that may be made by its manufacturer, is not guaranteed or endorsed by the publisher.
