# Supplementary material for: Nitrogen Supply Alters Rice Defense Against the Striped Stem Borer Chilo suppressalis
Source: Front Plant Sci. 2021 Jul 26;12:691292. doi: 10.3389/fpls.2021.691292 (PMC8351598; doi:10.3389/fpls.2021.691292)
Supplement: Supplementary file 1 [file Data_Sheet_1.pdf]

Supplementary Material

Nitrogen Supply Alters Rice Defenses Against the Striped Stem Borer *Chilo suppressalis*

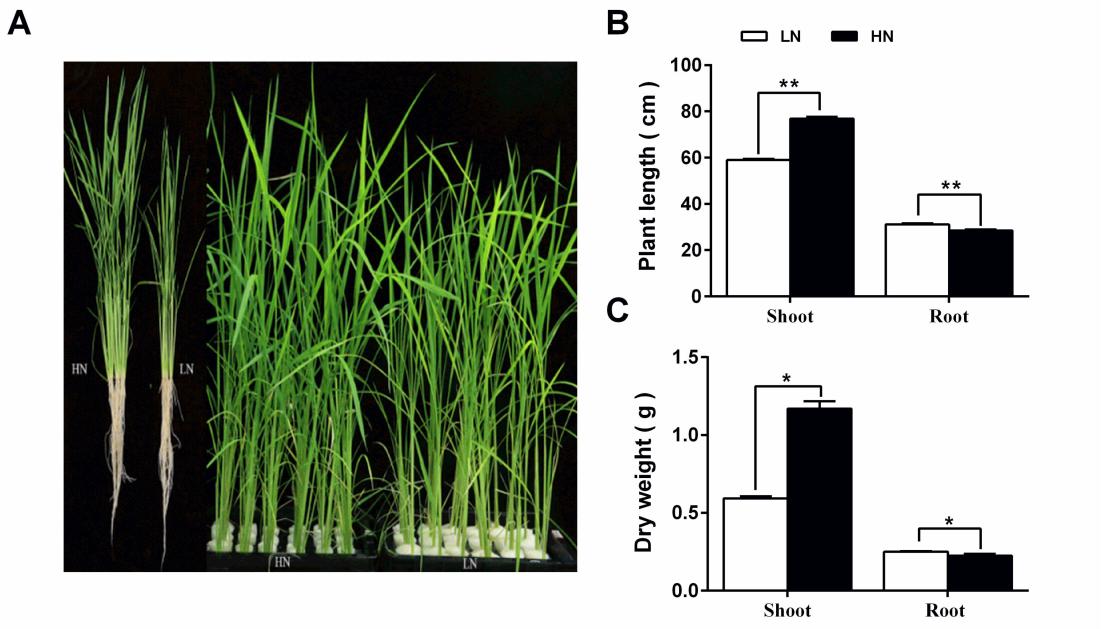

Supplementary Figure 1. Effect of nitrogen supply levels on rice growth. (A) Seedling morphology after nitrogen treatment. Seven-day-old seedlings were transplanted to nutrient solution containing 0.3 mM KNO_3 or 3 mM KNO_3 and cultured for other 30 days. Seedling morphology was taken. The height (B) and dry weight (C) of rice shoot and root were determined. Each data is the mean ± SE of twenty-two replicates. Asterisks (*) indicate a significant difference from the indicated samples *at * P < 0.05 or ** P < 0.01 by t-test.*

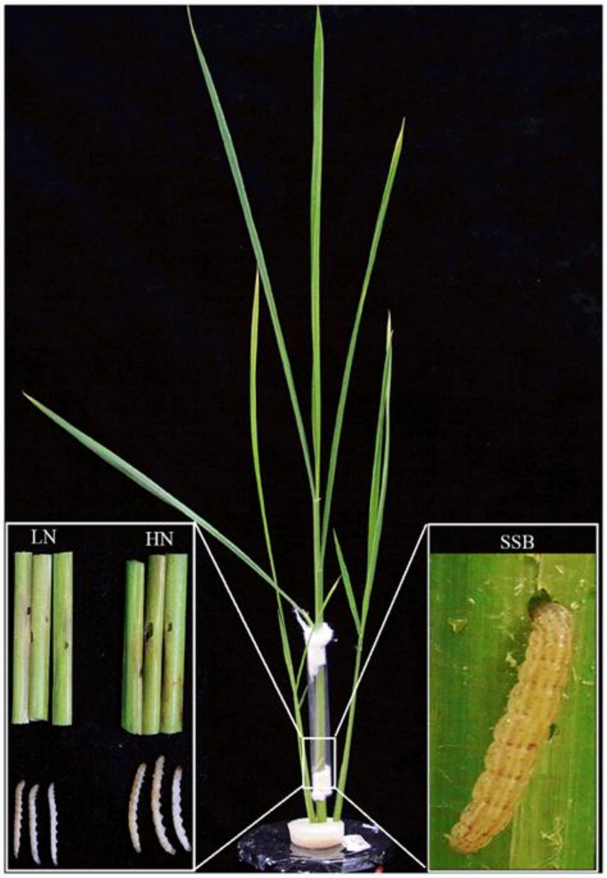

Supplementary Figure 2. SSB larval performance on stems of rice plants cultivated with different concentrations (0.3 and 3 mM) of nitrate. Seven-day-old seedlings were transplanted to nutrient solution containing 0.3 mM KNO_3 or 3 mM KNO_3 and cultured for other 40 days. The middle stem of rice was covered by one glass tube, then the top and bottom of tube was stuffed with cotton. SSB larvae at the third-instar stage was put into the glass tube. The morphology of rice stem and SSB larvae were taken at three days after SSB infestation.

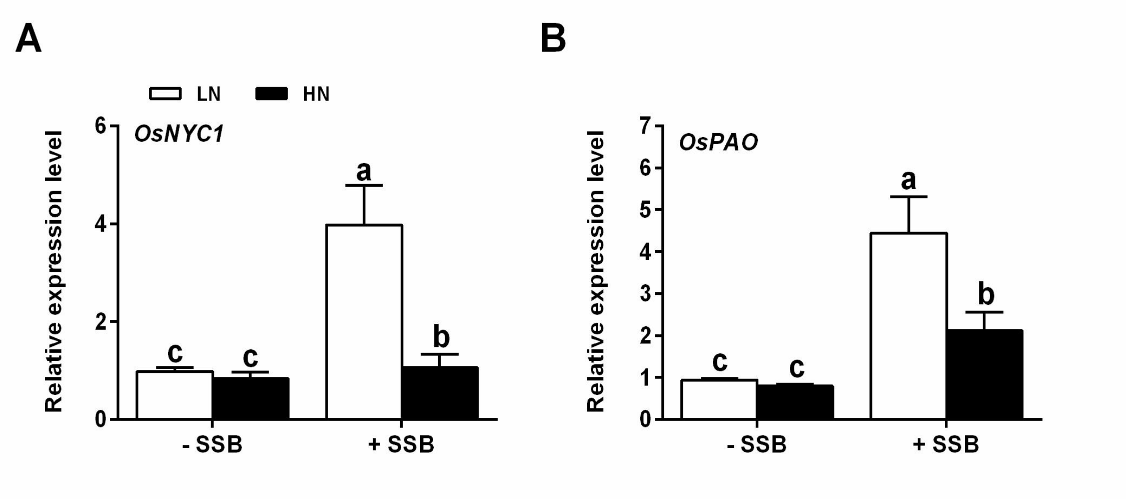

Supplementary Figure 3. Relative expression levels of genes involved in chlorophyll degradation in rice plants cultivated with different concentrations (0.3 and 3 mM) of nitrate and inoculated with the striped stem borer (SSB). The gene expression of *OsNYC1* (A) and *OsPAO* (B) among LN, HN, LNSSB and HNSSB treatment. LN, 0.3 mM KNO_3 cultured for 30 days; HN, 3 mM KNO_3 cultured for 30 days; LNSSB, 0.3 mM KNO_3 cultured for 30 days, then infected by SSB for 24 h; HNSSB, 3 mM KNO_3 cultured for 30 days, then infected by SSB for 24 h.

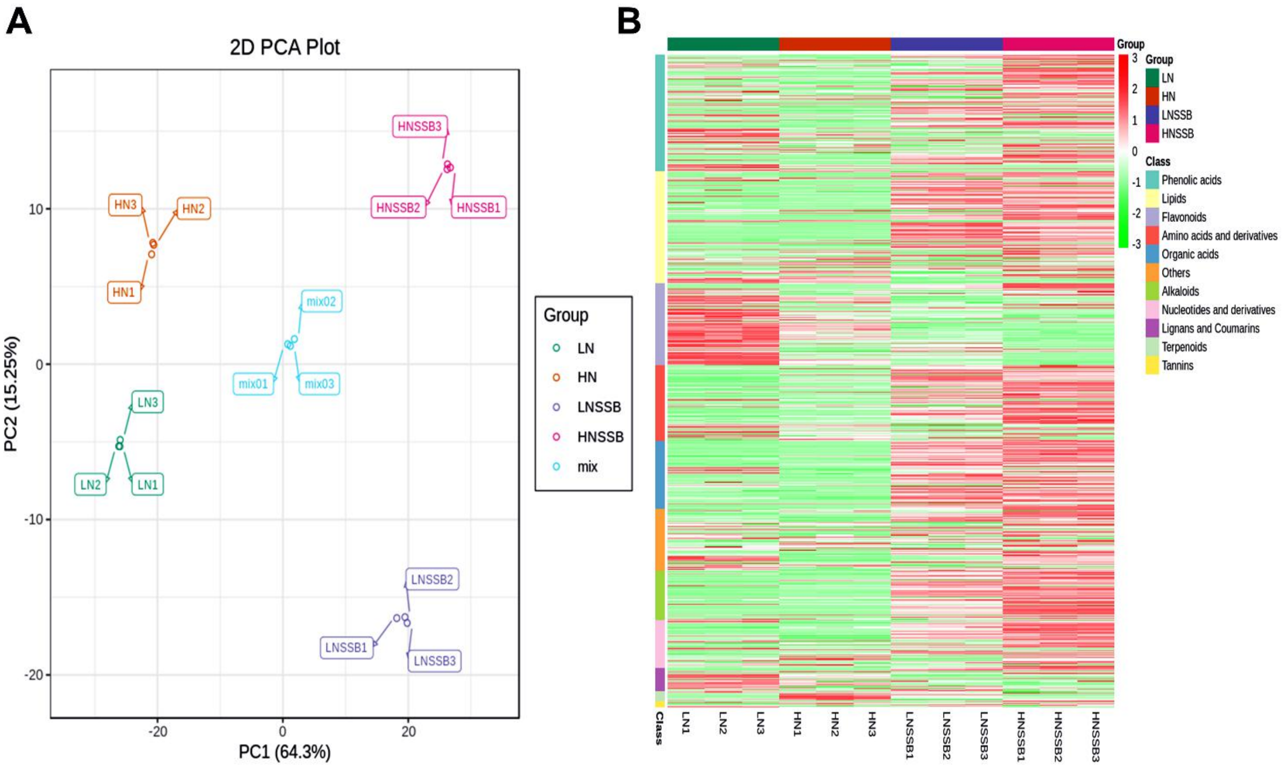

Supplementary Figure 4. The quality control for metabolome. (A) PCA analysis among LN, HN, LNSSB and HNSSB group. The abscissa and ordinate represent the scores of PC1 and PC2, respectively. mix: quality control sample prepared by mixing sample extracts. (B) Heatmap analysis of all detectable metabolites. LN, 0.3 mM KNO_3 cultured for 30 days; HN, 3 mM KNO_3 cultured for 30 days; LNSSB, 0.3 mM KNO_3 cultured for 30 days, then infected by SSB for 3 days; HNSSB, 3 mM KNO_3 cultured for 30 days, then infected by SSB for 3 days.

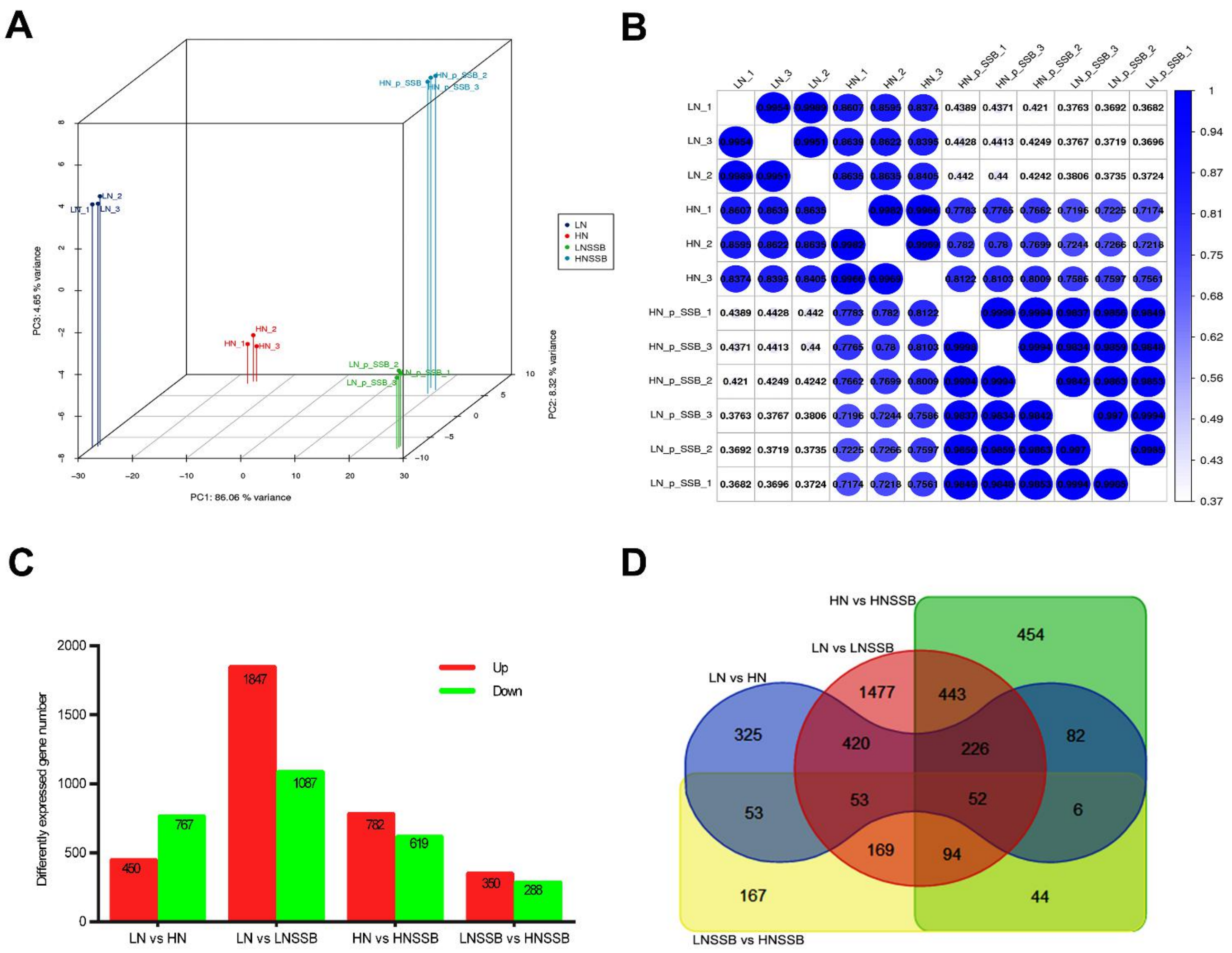

Supplementary Figure 5. Quality determination and differential gene analysis of RNA-Seq. The RNA-Seq metrics for quality control was shown as PCA analysis (A), correlation analysis (B) among LN, HN, LNSSB and HNSSB group. The overview of differential expressed gene under treatments was shown in (C). Venn diagram analysis of differential genes under treatment was shown in (D). LN, 0.3 mM KNO_3 cultured for 30 days; HN, 3 mM KNO_3 cultured for 30 days; LNSSB, 0.3 mM KNO_3 cultured for 30 days, then infected by SSB for 24 h; HNSSB, 3 mM KNO_3 cultured for 30 days, then infected by SSB for 24 h.

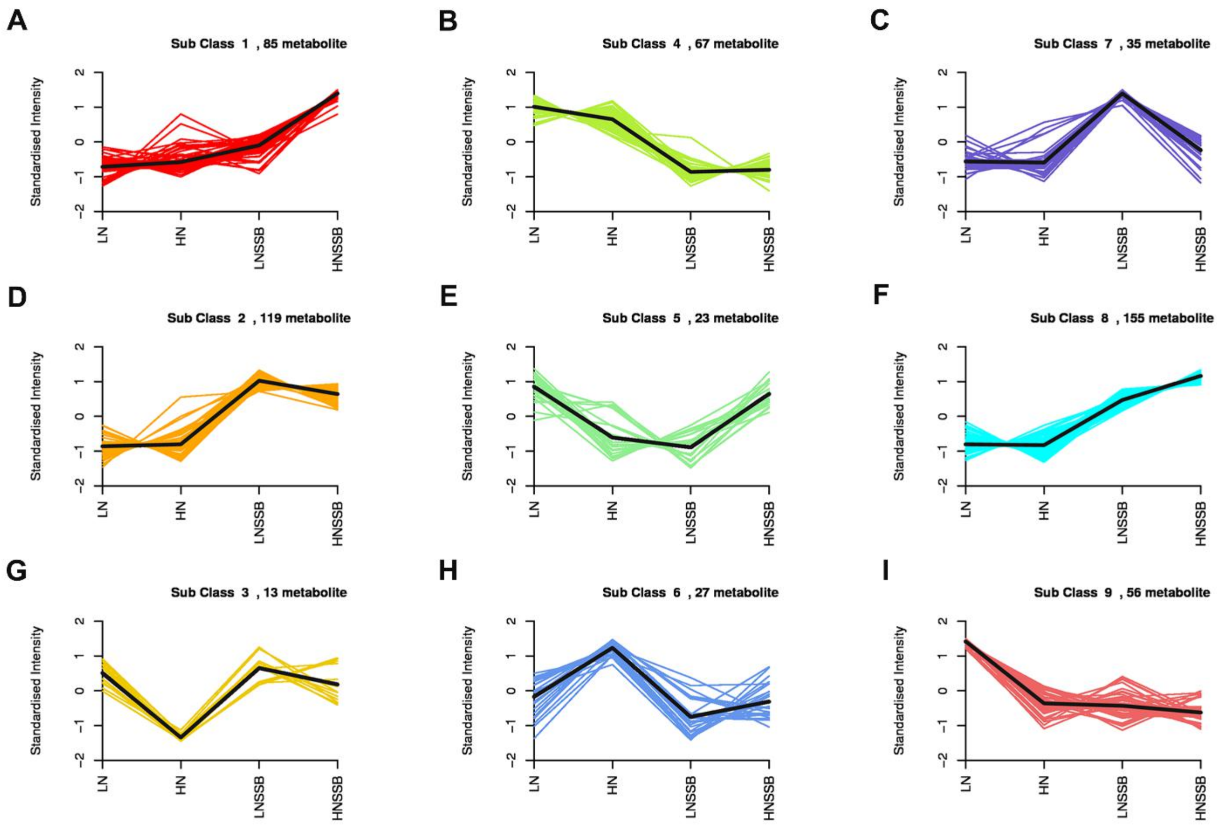

Supplementary Figure 6. K-means method clusters analysis. The x-axis shows different treatment, y-axis shows the relative content. LN, 0.3 mM KNO_3 cultured for 30 days; HN, 3 mM KNO_3 cultured for 30 days; LNSSB, 0.3 mM KNO_3 cultured for 30 days, then infected by SSB for 3 days; HNSSB, 3 mM KNO_3 cultured for 30 days, then infected by SSB for 3 days.

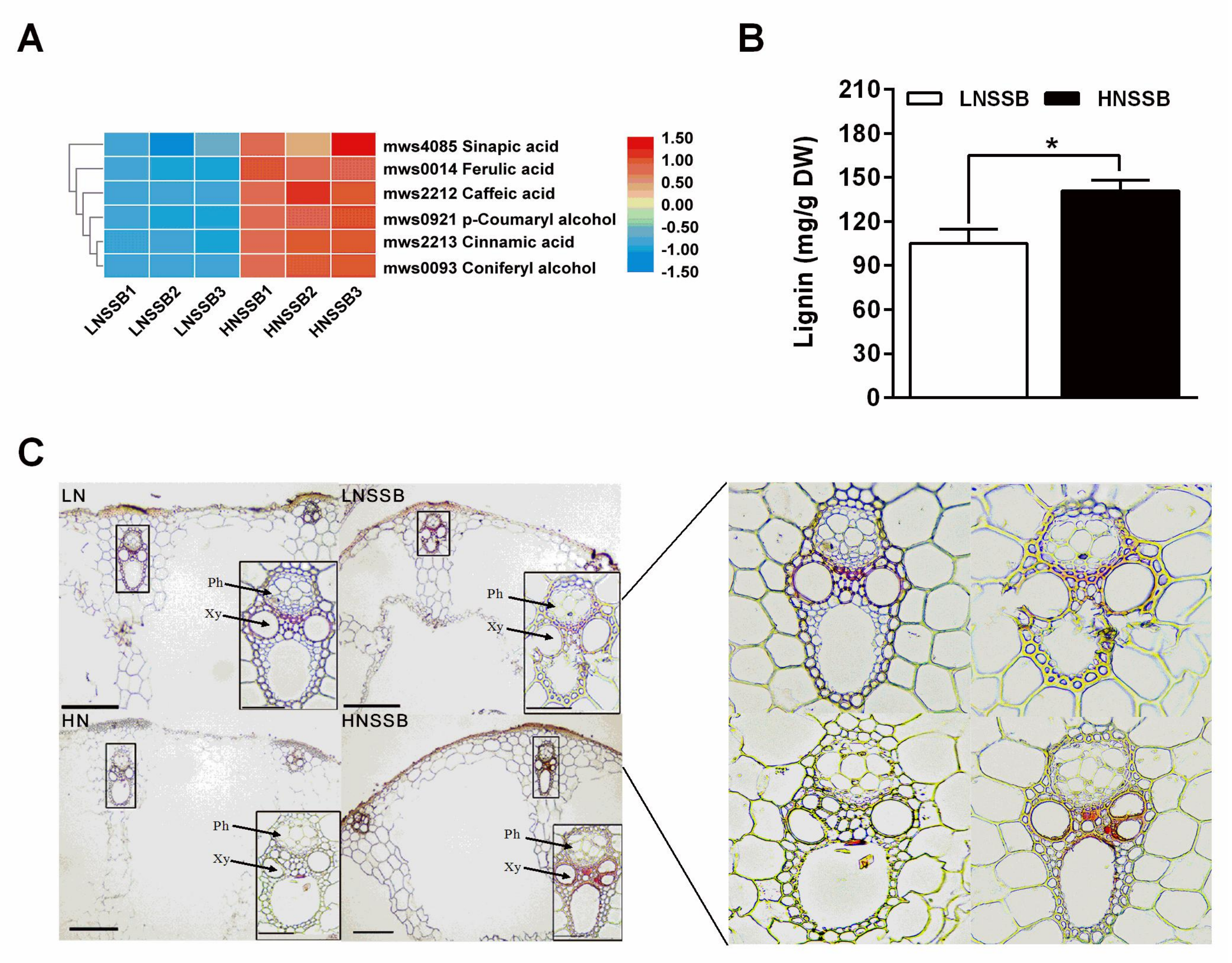

Supplementary Figure 7. The alteration of lignin content in the stems of rice plants cultivated with different concentrations (0.3 and 3 mM) of nitrate and inoculated with the striped stem borer (SSB). (A) The content of intermediate metabolites involved in lignin biosynthesis under treatment. (B) Quantitive evaluation of lignin content was taken at 72 h after SSB infestation. (C) The cell structure after phloroglucinol staining. Stem near the feeding site of 2 cm length was used for staining. Each stem was cut into slices horizontally of 101 μm thickness and then stained in 1 % (w/v) phloroglucinol liquor for 5 min, adding 18 % (v/v) HCl. The extent of staining was examined by a Leica microscope. The left side of (C) was enlarged in the right side of (C). LN, 0.3 mM KNO_3 cultured for 30 days; HN, 3 mM KNO_3 cultured for 30 days; LNSSB, 0.3 mM KNO_3 cultured for 30 days, then infected by SSB for 3 days; HNSSB, 3 mM KNO_3 cultured for 30 days, then infected by SSB for 3 days. Ph means phloem, Xy means xylem.

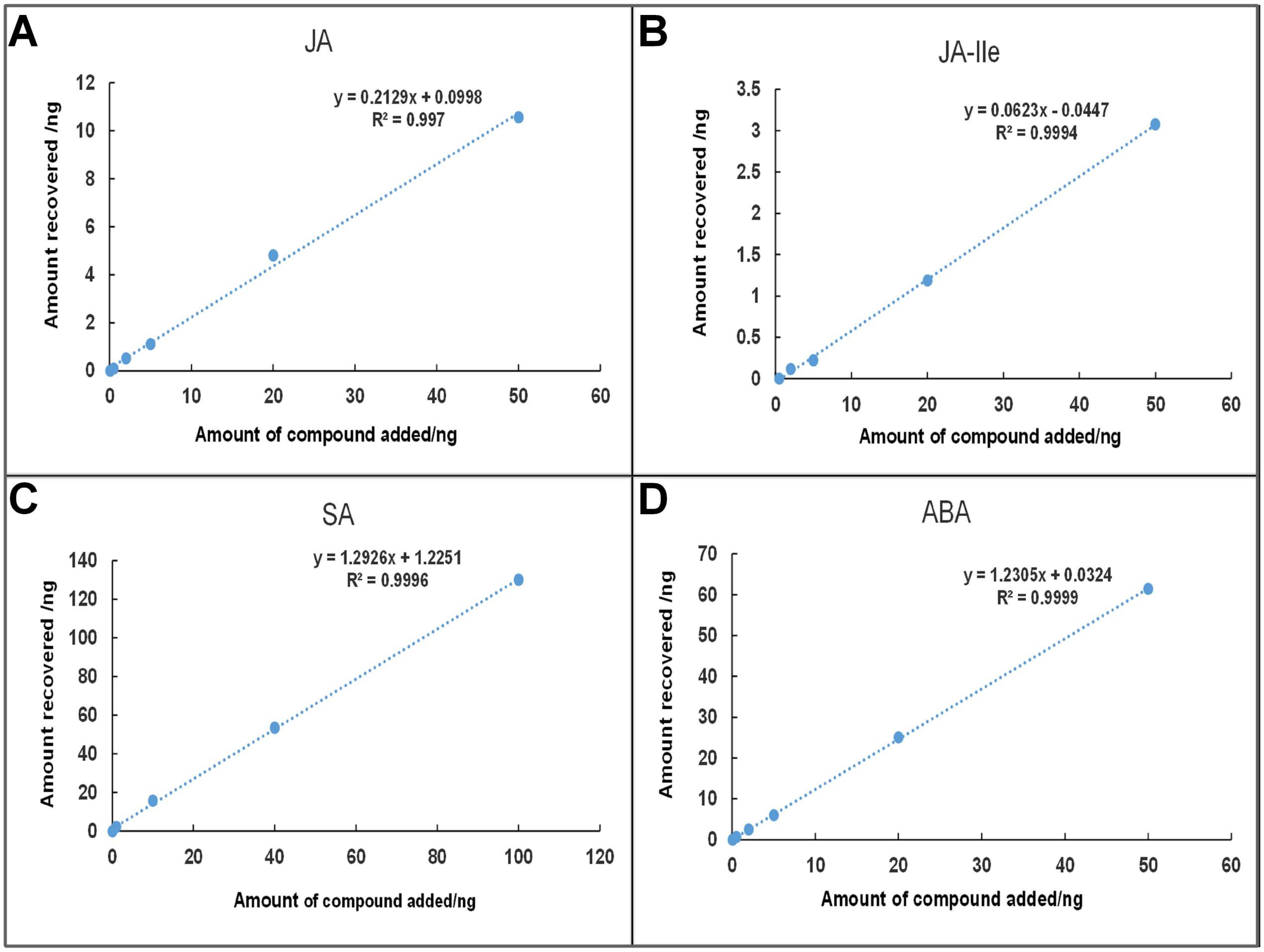

Supplementary Figure 8. Hormone quantitative standard curve. The standard curve of JA (A), JA-Ile (B), SA (C) and ABA(D) were taken by LC-MS analysis.

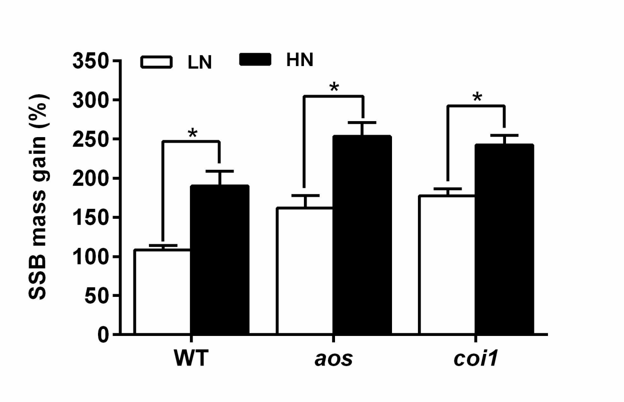

Supplementary Figure 9. Mass gain of the larvae of striped stem borer (SSB) fed on rice plants of WT, *aos* RNAi, *coi1* RNAi lines cultivated with different concentrations (0.3 and 3 mM) of nitrate. Seven-day-old seedlings were transplanted to nutrient solution containing 0.3 mM KNO_3 or 3 mM KNO_3 and cultured for other 40 days. Larvae at the third-instar stage were used for bioassays. The individual larvae were measured 3 d later, and the mean percentage of gain in mass was calculated. Values are mean ± SE (n=20). Asterisks (*) indicate Student's t-test significant at $P < 0.05$ versus the indicated samples.

Supplementary Table 1. Primers of genes involved in chlorophyll degradation for real-time qRT-PCR

Gene	Ensembl Gene ID	Sequence (5'-3')
OsActin-1	X15865.1	ctgacggagcgtggttac; ggaaggcgggaagaggac
OsNYC1	Os01g0227100	gaatccgtaattgggctgaa; ctggaagaggtccacctgag
OsPAO	Os03g0146400	gtgttgccttccactgtcct; actgaacatccgcaggaatc

Supplementary Table 2. Real time PCR of differentially expressed genes from nitrogen sufficient and nitrogen deficient under SSB infestation

	qPCR		RNA-Seq		
**Locus tag**	**Sequence (5'-3')**	**FC(LNSSB vs HNSSB)**	**FC(LNSSB vs HNSSB)**	**ρ-value**	**FDR**
**40612700**	gattctgttctgtggaatctgc<br>gttctcgaagccattgttacag	0.34	0.20	1.04E-02	4.10E-02
**100430200**	tagccagctagcaaccgacaa<br>ccgccggagcagtagttct	0.45	0.10	5.35E-04	3.20E-03
**20808800**	ctcatccgtggctaccacgtc<br>gggtaggacttcttggtgcc	0.38	0.14	7.25E-06	6.70E-05
**10374600**	gacgctaacaatgcgacaaata<br>cgggttctggaagctaacatta	0.67	0.08	2.64E-02	8.80E-02
**30273200**	gataaccctggtgtctggttc<br>aataaacgaatcacagccttgg	0.08	0.02	2.96E-07	3.56E-06
**70101000**	cagcatgaacaacgtctcg<br>tcaacgacatgtttctgttcac	0.55	0.04	4.94E-04	2.99E-03
**30368300**	gactaatgcaaacccatcgatc<br>gtagtacgtcatgtcgaatcga	7.57	9.15	3.59E-12	8.42E-11
**30368900**	cactggagttggcgtactac<br>catcacaaccctcgacaaag	0.11	0.13	3.90E-11	8.12E-10
**100109300**	ccaacgtcatcatgtcaaacat<br>cgcgcttaattaactatgcaca	0.15	0.14	5.75E-131	1.26E-127
